# How Social Determinants of Health Affect Outcomes Following Rotator Cuff Repair

**DOI:** 10.5435/JAAOSGlobal-D-25-00399

**Published:** 2026-01-02

**Authors:** Branden Wright, Jaynie X. Criscione, Vineeth Romiyo, Nicholas Pohl, Michael Curry, Krystal Hunter, Pietro M. Gentile, Lawrence S. Miller, Matthew T. Kleiner, Mark Pollard, Catherine J. Fedorka

**Affiliations:** From the Department of Orthopaedic Surgery, Cooper University Health Care, Camden, New Jersey (Dr. Wright, Ms. Criscione, Dr. Pohl, Dr. Curry, Mr. Gentile, Dr. Miller, Dr. Kleiner, Dr. Pollard, and Dr. Fedorka), and the Cooper Medical School of Rowan University, Camden, New Jersey (Dr. Romiyo, Dr. Hunter, Dr. Miller, Dr. Kleiner, Dr. Pollard, and Dr. Fedorka).

## Abstract

**Background:**

There has been an increased interest within orthopaedics investigating disparities in outcomes among populations of low socioeconomic status (SES). This study aims to investigate how SES, investigated by proxy through geocoding, affects postoperative outcomes following repair of rotator cuff tears.

**Methods:**

A total of 322 patients undergoing full-thickness rotator cuff tear repair (RCR) at a single institution from 2014 to 2021 were retrospectively reviewed and stratified by median household income and Social Deprivation Index (SDI) per zip code. Primary outcomes included patient-reported outcome measures (PROMs) at 12 months postoperatively. Multiple linear regression examined which variables correlated with American Shoulder and Elbow Surgeons (ASES) and Single Assessment Numeric Evaluation (SANE) scores.

**Results:**

The extremely/very low-income and high SDI patients had significantly decreased ASES, SANE, and satisfaction scores at 12 months postoperatively based on one-way analysis of variance (ANOVA) analysis. Multiple linear regression revealed that low- and medium-income groups had notable positive correlations with ASES and SANE scores. Highest SDI quartile, body mass index, female sex, and current smoking status had notable negative correlations with PROMs.

**Conclusion:**

Based on ANOVA, patients from areas of lowest income and highest SDI had lower PROMs following RCR, although multiple regression demonstrates that PROMs are multifactorial. The results should be interpreted in the context of current literature and used to raise awareness among orthopaedic surgeons of risk factors affecting PROMs following RCR. As health care trends toward patient satisfaction-driven financial models, we stress the importance of risk-adjusted compensation and performance models for orthopaedic surgeons performing RCRs and preoperative expectation counseling.

Research into disparities within the field of orthopaedics is garnering substantial interest.^1^ For example, utilization rates of total joint arthroplasties have been considerably lower for minority populations since the 1990s.^2^ Although other surgical specialties have decreased the gap in utilization of their respective procedures in minority populations, the gap for major orthopaedic procedures has widened during the 21st century.^3^ This is complicated by the persistently low awareness of disparities among orthopaedic surgeons.^4^ In recent years, research efforts on disparities in orthopaedic care have expanded within the subspecialties of joint reconstruction, spine, and trauma.^5,6^ However, research remains limited surrounding disparities in outcomes following rotator cuff repairs (RCR).

Approximately 4.5 million visits can be attributed to rotator cuff pathology per year, and 200,000 to 300,000 of these cases undergo surgical intervention, with an increasing incidence.^7,8^ Despite being a common procedure, no large studies have investigated disparities in patient-reported outcome measures (PROMs) following RCR for populations of low socioeconomic status (SES). In addition to a growing incidence, circumstances common to individuals of low SES are potential barriers to equitable outcomes following RCR. For example, individuals with Medicaid insurance have difficulty accessing initial orthopaedic care compared with those with private insurance, possibly delaying diagnosis and treatment.^8^ For those who can access care, research indicates that minority patients and patients from low-income areas are more likely to be treated by low-volume facilities and surgeons, which negatively affects outcomes.^9^ Following surgical repair, it has been reported that fewer physical therapy practices accept patients with Medicaid as compared with private insurance following RCR.^10,11^ Owing to the high incidence of rotator cuff injuries and known potential barriers to equitable outcomes, investigations into outcome disparities after RCR are warranted to ensure equitable care is being delivered.

The multifactorial nature of health disparities makes it difficult for researchers to agree upon a universal method for investigation. Further complicating this, determinants of SES, such as household income, are sensitive details to discuss and collect. There are several individual- and community-level parameters that can be used as a proxy to assess for SES. In the joint reconstruction literature, studies have conducted investigations based on race, insurance status, net worth, median income per zip code, and zip code–based social deprivation.^12-15^ Recent literature has pointed toward geocoding, which is grouping patients based on metrics tied to their home zip code, as a proxy for SES. Previous studies have published data based on geocoding the median household income of zip codes to assess SES in the absence of individual-level information, although with certain limitations.^12,16,17^ In addition to geocoding based on median income, the Social Deprivation Index (SDI) is a variable that can be geocoded to capture the complex nature of SES and can help strengthen the validity of community-level SES stratifications. SDI is a validated metric that incorporates an area's average income, education, employment, housing, household characteristics, transportation, and demographics to formulate an overall quantification of a certain area's social deprivation and healthcare needs.^18^ SDI has been shown to be useful in orthopaedics.^19^ Together, median household income per zip code and SDI can be used to geocode research subjects and stratify individuals into discrete levels of SES.

The purpose of our study was to investigate potential disparities in outcomes after RCR of full-thickness tears using median household income per zip code and SDI as measures of SES. We also sought to investigate which factors and comorbidities influenced PROMs. We hypothesized that individuals from areas with lower median household income and high SDI would report lower PROMs 1 year after RCR compared with individuals from areas with higher median income and lower SDI.

## Methods

We conducted a retrospective chart review, using the International Classification of Diseases (ICD) 10 codes M75.XX, S46.XX and current procedural terminology (CPT) code 29827 to identify patients who underwent arthroscopic RCR from January 2014 to December 2020. The study protocol was approved by our institution's Institutional Review Board. All patients were treated by one of the four fellowship trained orthopaedic surgeons at our institution. Individual charts were screened to include patients with a full-thickness tear documented in the surgical note and a current New Jersey, Pennsylvania, or Delaware address. Full-thickness tears were found based on the operative surgeon's surgical note describing a full-thickness tear. Excluded were patients with surgical notes specifying that the tear was partial thickness or did not explicitly report that it was full thickness.

**Figure 1:**
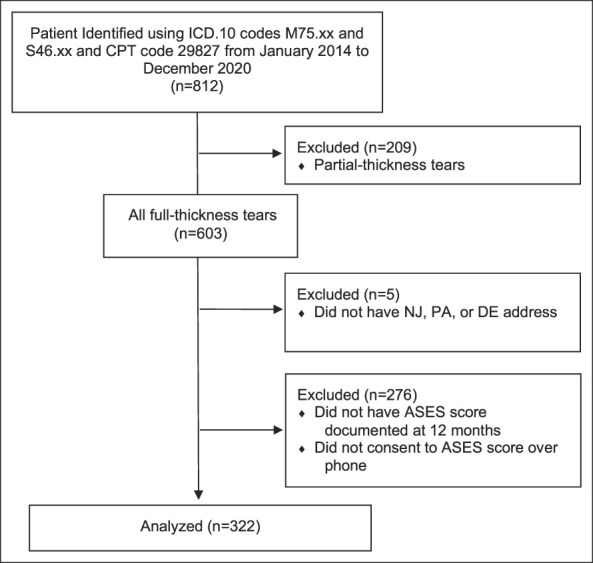
Flow chart demonstrating patient inclusion/exclusion.

Primary outcomes included the American Shoulder and Elbow Surgeons (ASES) survey, with scores range from 0 to 100, with 0 indicating a worse shoulder condition and 100 indicating a better shoulder condition; Single Assessment Numeric Evaluation (SANE) score representing function as a percentage of normal; and patient satisfaction scores.^20,21^ ASES scores were obtained for the repaired shoulder. SANE scores were obtained for the repaired shoulder compared with normal function before injury and compared with their contralateral shoulder. Charts were examined for preoperative and postoperative ASES and SANE scores. Unfortunately, chart review revealed only 57 patients with preoperative scores, but enough to include a small statistical analysis. Primary outcomes were the postoperative ASES and SANE scores recorded at 12 months as maximal functional improvement following RCR is expected at 12 months and stabilizes thereafter.^22^ Patients without an ASES score recorded at 12 months were contacted through telephone to consent to participate in the study. Once consent was received, the research team obtained ASES, SANE, and satisfaction scores. After this, only patients with ASES scores recorded were considered for further analysis (n = 322; Figure 1). Secondary outcome measures included patient return to work status, age, body mass index (BMI) at the time of surgery, race, sex, Charlson Comorbidity Index (CCI), smoking status at the time of surgery, number of tendons involved, tear size, time from injury to treatment, complications, retear rate, and repeat RCR rate.

The patients were geocoded to create the final cohorts: Patients were stratified into four income groups defined as extremely low/very low income (EVL), low income, middle income, and high income by analyzing the median household income per subject zip code and SDI. Median household income data were obtained from the United States Census Bureau Quickfacts search tool.^23^ Income classifications were defined by the Federal Reserve and Federal Department of Housing and Urban Development definitions for the Philadelphia metropolitan area.^16,24,25^ Refer to Table 1 for income definitions. SDI values according to zip code were taken from the most recent publicly available data.^26^ SDI values were stratified based on quartiles, with higher values representing lower SES.

**Table 1:** Income Definitions

Income Definition^a^	Range
Extremely low	<$31,600
Very low	$31,601-$52,700
Low income	$52,701-$84,300
Median income for Philadelphia Metro area	$105,400
Middle income	$84,301-$126,480
High income	>$126,480

a Income limits according to Federal Reserve and Federal Housing and Urban Development Agency.^27^

One-way analysis of variance (ANOVA) statistical analysis was used to examine differences between the four income groups and between SDI groups as separate independent variables against dependent variables. Dependent variables examined by ANOVA include preoperative ASES and SANE scores, 12-month postoperative ASES and SANE scores, 12-month ASES and SANE change, BMI, CCI, number of tendons involved, time to treatment, and tear size. Chi squared tests were performed to detect differences between income groups and SDI quartile groups for sex, satisfaction scores, race, smoking status, and return to work rate. Multiple regression analyses were performed to identify notable factors correlating with ASES and SANE scores among the variables of age, sex, BMI, CCI, number of tendons involved, and smoking status. Multiple regressions were performed separately for SDI and median income as dependent variables because of the high interrelatedness between the two.

## Results

For primary outcomes stratified by median income, EVL income patients reported lower postoperative ASES scores at 12 months (*P* = 0.017; Table 2). Likewise, EVL income groups reported lower postoperative SANE scores at 12 months (*P* = 0.012). Satisfaction scores were significantly lower for EVL income (*P* = 0.008). No significant difference was observed in preoperative ASES and SANE scores for those available nor in 12-month postoperative ASES and SANE increases from baseline among median income groups (Table 3).

**Table 2:** Primary and Secondary Outcomes Results

Factor or Variable	Median Household Income				*P* Value
	EVL	Low	Middle	High	
N = 332	65	147	84	36	
Sex (F)^a^ (%)	53.8	44.9	53.6	44.4	0.458
Race^a^ (%)					
White	17.9	42.9	42.9	52.8	<0.001^b^
Black	41.8	13.6	11.9	8.3	
Asian	0.0	1.4	2.4	0.0	
Hispanic	22.4	4.8	4.8	2.8	
Other	17.9	37.4	38.1	36.1	
Age^c^	56.6 ± 8.2	58.6 ± 8.9	58.5 ± 8.6	62.0 ± 8.5	0.024^b^
Smoking status^a^ (%)					
None	53.8	53.1	66.7	69.4	0.120
Current	27.7	21.1	15.5	8.3	
Former	18.5	25.9	17.9	22.2	
BMI^c^	31.1 ± 5.9	31.2 ± 6.0	31.9 ± 7.6	27.4 ± 4.9	0.002^b^
CCI^c^	3.05 ± 1.55	3.32 ± 1.88	3.04 ± 1.59	3.61 ± 1.57	0.123
Tendons involved^a^ (%)					0.181
<2	41.5	54.4	47.6	61.1	
≥2	58.5	45.6	52.4	38.9	
Tear size (cm)^c^	2.43 ± 1.17	2.20 ± 1.24	2.22 ± 1.42	2.88 ± 1.67	0.109
Return to work^a^ (%)	65.5	82.8	91.1	79.4	0.002^b^
ASES^c^	72.8 ± 27.2	81.0 ± 21.9	84.8 ± 19.5	85.0 ± 20.0	0.017^b^
SANE ipsilateral^c^	76.4 ± 25.1	82.5 ± 20.0	87.4 ± 18.0	82.9 ± 22.7	0.012^b^
SANE contralateral^c^	74.1 ± 29.5	82.5 ± 22.3	86.7 ± 19.8	83.7 ± 25.5	0.021^b^
Patient satisfaction^a^ (%)					
Extremely satisfied	44.1	74.3	73.2	58.3	0.008^b^
Satisfied	33.9	12.5	17.1	27.8	
Somewhat satisfied	11.9	8.8	6.1	8.3	
Not satisfied at all	10.2	4.4	3.7	5.6	
Time to treatment (days)^c^	283.5 ± 312.2	225.0 ± 232.6	248.2 ± 314.8	378.0 ± 407.3	0.046^b^

Factor or Variable	SDI Quartiles				*P* Value
	1	2	3	4	
N = 332	92	78	67	94	
Sex (F)^a^ (%)	51.1	48.7	38.8.0	54.3	0.263
Race^a^ (%)					
White	46.7	39.7	41.2	29.8	<0.001^a^
Black	5.5	12.8	19.1	33.0	
Asian	1.1	3.8	0.0	0.0	
Hispanic	5.4	5.1	2.9	16.0	
Other	41.3	38.5	36.8	21.3	
Age^c^	60.1 ± 9.4	58.6 ± 7.3	58.6 ± 7.3	57.0 ± 7.9	0.152
Smoking status^a^ (%)					
None	59.8	65.4	55.2	53.2	0.124
Current	15.2	21.8	14.9	25.5	
Former	25.0	12.8	29.9	21.3	
BMI^c^	29.5 ± 6.5	31.2 ± 6.6	31.4 ± 6.7	31.8 ± 6.0	0.024^b^
CCI^c^	3.47 ± 1.85	2.94 ± 1.41	3.15 ± 1.62	3.24 ± 1.84	0.338
Tendons involved^a^ (%)					0.257
<2	58.7	44.9	52.2	46.8	
≥2	41.3	55.1	47.8	53.2	
Tear size (cm)^c^	2.48 ± 1.49	2.14 ± 1.43	2.20 ± 1.29	2.41 ± 1.12	0.112
Return to work^a^ (%)	84.3	88.9	85.2	68.3	0.005^b^
ASES^c^	84.9 ± 20.0	80.7 ± 21.2	85.1 ± 19.0	73.5 ± 26.9	0.014^b^
SANE ipsilateral^c^	85.8 ± 19.4	81.6 ± 22.7	87.5 ± 13.3	77.1 ± 24.7	0.023^b^
SANE contralateral^c^	86.9 ± 21.9	80.14 ± 24.1	84.70 ± 19.8	76.32 ± 27.3	0.022^b^
Patient satisfaction^a^ (%)					
Extremely satisfied	69.6	75.0	74.6	48.8	0.006^b^
Satisfied	19.6	9.7	20.6	27.4	
Somewhat satisfied	7.6	11.1	1.6	13.1	
Not Satisfied at all	3.3	4.2	3.2	10.7	
Time to treatment (days)^c^	276.5 ± 311.6	282.6 ± 368.5	240.8 ± 264.9	236.5 ± 237.5	0.681

ASES = American Shoulder and Elbow Surgeons score, BMI = body mass index, CCI = Charlson Comorbidity Index, EVL = extremely/very low income, SANE = Single Assessment Numeric Evaluation Score, SDI = Social Deprivation Index, Std Beta = standard beta, 95% CI = 95% confidence interval

a Statistics performed via chi square test.

b Statistical significance (*P* < 0.05).

c Statistics performed via one way ANOVA test.

**Table 3:** Preoperative American Shoulder and Elbow Surgeons and Single Assessment Numeric Evaluation Scores

Factor or Variable	Median Household Income Categories				*P* Value
	EVL	Low	Middle	High	
N	12	27	13	5	
Preoperative ASES^a^	34.06 ± 19.87	31.42 ± 18.78	37.91 ± 18.39	27.79 ± 15.15	0.680
Preoperative SANE^a^	38.55 ± 22.33	28.63 ± 20.01	36.64 ± 21.02	37.60 ± 17.07	0.486
12-month ASES change^a^	45.41 ± 22.82	56.91 ± 18.14	33.86 ± 21.75	38.36 ± 16.92	0.061
12-month SANE change^a^	39.90 ± 28.47	61.67 ± 22.00	40.80 ± 33.20	36.00 ± 23.38	0.086

Factor or Variable	SDI Quartiles				*P* Value
	1	2	3	4	
N	12	12	13	20	
Preoperative ASES^a^	33.68 ± 16.62	31.96 ± 23.47	33.18 ± 17.85	33.49 ± 17.97	0.996
Preoperative SANE^a^	33.00 ± 16.30	34.00 ± 25.48	30.55 ± 18.73	34.89 ± 21.95	0.957
12-month ASES change^a^	45.37 ± 18.43	41.61 ± 32.42	48.15 ± 14.13	48.72 ± 21.80	0.898
12-month SANE change^a^	51.10 ± 22.76	40.67 ± 37.45	57.67 ± 22.10	47.50 ± 29.06	0.640

ASES = American Shoulder and Elbow Surgeons score, SANE = Single Assessment Numeric Evaluation Score, SDI = Social Deprivation Index, EVL = extremely/very low

a Statistics performed using Kruskal-Wallis and reported as mean rank values.

For primary outcomes stratified by SDI, fourth quartile SDI patients reported significantly decreased postoperative ASES scores at 12 months (*P* = 0.014; Table 2). For the fourth quartile SDI group, SANE scores were reported as lower (*P* = 0.023) and satisfaction scores were significantly lower (*P* = 0.006). No significant differences were observed in preoperative ASES and SANE scores for those available nor in 12-month postoperative ASES and SANE changes from baseline among SDI quartiles (Table 3).

Multiple linear regression with ASES revealed low- and middle-income groups had a positive correlation with ASES scores. Female sex, BMI, current smoking status, and CCI had a negative correlation with ASES scores (Table 4). For SANE scores, medium income had a positive correlation, while BMI and current smoking status had negative correlations (Table 4).

**Table 4:** Multiple Regression Models for American Shoulder and Elbow Surgeons and Single Assessment Numeric Evaluation Scores With Median Income

Variable	ASES			SANE		
	Std Beta	95% CI	*P* Value	Std Beta	95% CI	*P* Value
Income classification						
Very low	Reference					
Low	7.00	0.56 to 13.45	0.034^a^	5.31	−0.94 to 11.57	0.097
Medium	11.18	4.07 to 18.28	0.002^a^	10.50	3.66 to 17.35	0.003^a^
High	5.82	−3.37 to 15.00	0.216	1.57	−7.29 to 10.42	0.729
Age	0.23	−0.12 to 0.58	0.193	0.22	−0.12 to 0.56	0.213
BMI	−0.128	−1.12 to −0.33	<0.001^a^	−0.60	−0.97 to −0.22	0.002^a^
Sex-female	−7.25	−12.04 to −2.45	0.003^a^	−3.09	−7.71 to 1.53	0.191
CCI	−1.75	−3.44 to −0.07	0.043^a^	−1.16	−2.87 to 0.55	0.183
≥ 2 tendons	−1.48	−6.26 to −3.29	0.543	−0.27	−4.87 to 4.32	0.907
Smoking status						
Nonsmoker	Reference					
Current smoker	−11.62	−17.92 to −5.32	<0.001^a^	−9.30	−15.43 to −3.16	0.003^a^
Former smoker	−0.88	−6.84 to 5.09	0.774	−1.29	−7.01 to 4.43	0.659

ASES = American Shoulder and Elbow Surgeons score, BMI = body mass index, CCI = Charlson Comorbidity Index, SANE = Single Assessment Numeric Evaluation Score, Std Beta = standard beta, SDI = Social Deprivation Index, 95% CI = 95% confidence interval

a Statistically significant (*P* < 0.05).

Multiple regression with ASES revealed that fourth quartile SDI, BMI, female sex, and current smoking status negatively affected ASES scores (Table 5). Only BMI and current smoking status had a notable correlation with SANE scores.

**Table 5:** Multiple Regression Models for American Shoulder and Elbow Surgeons and Single Assessment Numeric Evaluation Scores With Social Deprivation Index

Variable	ASES			SANE		
	Std Beta	95% CI	*P* Value	Std Beta	95% CI	*P* Value
SDI classification						
First quartile	Reference					
Second quartile	−2.92	−9.64 to 3.79	0.394	−2.78	−9.24 to 3.68	0.400
Third quartile	0.81	−6.17 to 7.79	0.820	2.47	−4.27 to 9.21	0.474
Fourth quartile	−7.86	−14.34 to −1.37	0.018^a^	−5.92	−12.18 to 0.34	0.065
Age	0.22	−0.13 to 0.57	0.213	0.19	−0.15 to 0.54	0.268
BMI	−0.63	−1.12 to −0.24	0.002^a^	−0.49	−0.86 to −0.11	0.012^a^
Sex-female	−6.88	−11.70 to −2.06	0.006^a^	−2.59	−7.24 to 2.06	0.277
CCI	−1.72	−3.43 to 0.00	0.051	−1.07	−2.82 to 0.66	0.225
≥2 tendons	−1.39	−6.18 to 3.79	0.569	−0.14	−4.75 to 4.46	0.951
Smoking status						
Nonsmoker	Reference					
Current smoker	−11.46	−17.75 to −5.17	<0.001^a^	−8.92	−15.06 to −2.78	0.005^a^
Former smoker	−1.34	−7.35 to 4.67	0.663	−1.91	−7.69 to 3.84	0.516

ASES = American Shoulder and Elbow Surgeons score, BMI = body mass index, CCI = Charlson Comorbidity Index, SANE = Single Assessment Numeric Evaluation score, Std Beta = standard beta, SDI = Social Deprivation Index, 95% CI = 95% confidence interval

a Statistically significant (*P* < 0.05).

For secondary outcomes, no statistical difference was noted between sex, smoking status, CCI, number of tendons involved, or tear size between groups regardless of stratification by income or SDI (Table 2). Stratified by median income, patients from EVL income areas were significantly younger than other income groups (*P* = 0.024). Patients from EVL, low-income, and middle-income groups had higher average BMI compared with the high-income group (*P* = 0.002). When stratifying by both median income and SDI quartile, EVL and high SDI groups were majority African American and Hispanic (*P* < 0.001). Extremely low/very low income (*P* = 0.002) and high SDI (*P* = 0.005) areas had a lower rate of return to work. The high-income group stratified by median income had a longer time to treatment (*P* = 0.046); however, this was not statistically significant when stratified by SDI.

## Discussion

One way ANOVA analysis supported the hypothesis that individuals from areas of EVL income and high SDI would report lower ASES and SANE scores compared with those of higher-income groups and lower SDI. When comparing median income versus SDI, most outcomes had similar trends and statistical results, demonstrating reliability between the two variables assessing for level of SES. However, this information should be viewed in the appropriate context, as the multiple regression showed that many factors correlated with functional outcome scores. When compared with EVL, low- and medium-income groups had positive correlations with functional outcome scores. When compared with first quartile SDI or the least deprived, the fourth quartile SDI had a negative correlation with functional outcome scores. In both multiple regressions, BMI, female sex, and current smoking status had negative correlations with functional outcome scores. CCI was negatively correlated to ASES in the regression with income classification. Each of the factors included in this study's multiple regression analysis supports the published literature.

There is ample evidence that populations with low SES have higher rates of smoking and obesity, both of which negatively affect healing after RCR.^28–32^ Research also reports that female sex can negatively influence PROMs following RCR.^33^ With these results, this study supports the concept that low SES plays a complicated, multifactorial role in all facets of health outcomes.^34^ Therefore, orthopaedic surgeons should be aware of these modifiable (BMI, smoking) and nonmodifiable (SES, sex) risk factors when counseling patients for RCR.

In addition, a lower return to work rate was observed in our EVL income and high SDI groups that may be explained by previous studies. Individuals of low SES have a higher likelihood of working a physically demanding job, and increasing work intensity leads to a lower chance of returning to work following RCR.^35,36^ Higher unemployment following RCR may imply a change in insurance status, which also has been shown to influence outcomes following RCR.^34^

This study found lower satisfaction scores for patients in the EVL income group and highest SDI quartile. For patients of low SES with multiple risk factors, preoperative counseling is important to establish realistic expectations and address modifiable risk factors before surgery. Studies have shown a strong correlation between preoperative patient expectations and postoperative patient satisfaction.^27,37^ Evidence suggests that patients of low SES benefit equally from RCR; however, their preoperative baseline functional status tends to be lower than individuals of high SES, leading to perceived worse outcomes.^38^ Therefore, it is important for clinicians to assess patients' understanding of the goals of RCR and how risk factors may influence their outcomes. Orthopaedic surgeons can then tailor their preoperative counseling to each patient while addressing risk factor mitigation. For example, smoking cessation programs before arthroplasty procedures are effective at reducing complications and are cost efficient.^39^ Thus, surgeons performing RCR have a role in influencing patient satisfaction and PROMs through expectation counseling. Decreasing the impact of modifiable risk factors is imperative to prevent barriers to treatment and favorable outcomes for low SES populations.

Our results did not show SES as an independent risk factor, but we are concerned that lower functional and satisfaction scores in patients of low SES, especially with multiple risk factors, may exacerbate the mechanisms driving the disparities. As health care moves toward patient outcome and satisfaction-based compensation systems, we want to emphasize two points to ensure patients of low SES with multiple risk factors are not discriminated against as candidates for RCR. First, we want to emphasize the need for risk-adjusted compensation and performance models. Studies in spine and joint reconstruction call for risk adjustment payment models with Medicare reimbursement because research has shown that patients of low SES have greater resource utilization, higher complications rates, and higher readmission rates after surgery.^6,40^ Therefore, surgeons may defer from participating in the care of these patients because of worry over adequate compensation. Supporting risk adjustment models, a study using a database of surgeons performing RCR found a wide variation of risk-adjusted 6-month ASES scores compared with unadjusted scores, with reported surgeon average ASES variations between −13.8 to 10.3.^38^ The study indicates surgeons are improperly evaluated when they treat patients of lower SES under current models, resulting in inequitable financial losses. The authors of this study agree that risk-adjusted payment and performance models for RCR are necessary to alleviate provider hesitation secondary to adequate compensation. Establishing proper provider compensation can help mitigate issues accessing orthopaedic care for individuals of low SES with multiple risk factors requiring RCR.

This study has several limitations, including having a small number of preoperative baseline functional scores, using community-level assessments of SES, a sample population that was limited by enrollment, and a potential for sampling bias because of missing follow-up data and ICD-10/CPT codes. Although there was no difference in a small sample of preoperative ASES and SANE scores, having preoperative baseline functional scores for our entire cohort would have increased the power of evidence regarding whether lower baseline functionality influenced satisfaction and PROMs. Asking patients to retrospectively report baseline functional assessments after an intervention has taken place is known to introduce notable negative recall bias, so this was not part of our study design.^41^ An inherent limitation of our study was using median income per zip code and SDI per zip code as proxies for SES. Although there is evidence to support the use of geocoding, it should be interpreted cautiously as its validity substituting for individual level SES is not 100% certain.^17^ The sample size of 322 patients is the largest study to date investigating patient reported outcomes following RCR based on SES. However, the enrollment was limited to 53% of the original sample because of incomplete data. Large multicenter studies with more enrolled patients that capture multiple regions are needed to further validate our observed results. Sampling bias cannot be ruled out, as our inclusion criteria was contingent on those who had postoperative ASES scores. Perhaps those with worse outcomes were unwilling to participate, or individuals from low-income areas without stable housing were unable to be contacted. Relying on ICD-10/CPT codes may have influenced those included and excluded if the ICD-10/CPT coding was incorrect. Considering these significant limitations, the results of this study should be seen as providing support to recent literature about modifiable and nonmodifiable factors that can influence RCR.

Future studies should prospectively compare both preoperative and postoperative PROMs after RCR to examine outcomes more precisely from baseline. Future research should also focus on functional outcomes at different time intervals to evaluate disparities in recovery time, which is especially important for individuals needing to return to work. Finally, further investigations into risk-adjusted compensation and performance models for orthopaedic surgeons performing RCR should be explored.^38^

## Conclusion

Individuals from EVL income and higher SDI areas had worse ASES, SANE, and satisfaction scores compared with individuals from higher income and lower SDI areas with a one-way ANOVA analysis. However, multiple regression analysis revealed that several factors influenced ASES and SANE scores in addition to median income and SDI per zip code. Other factors found to influence patient-reported outcomes include sex, BMI, and current smoking status. Given the limitations and factors influencing patient-reported outcomes, these results should be taken into appropriate context. This study supports current literature about modifiable (BMI, smoking status) and nonmodifiable (SES, sex) factors influencing PROMs following RCR. Orthopaedic surgeons should be cognizant of these factors when treating individuals requiring RCR and recognize geocoding as a validated tool to use as a substitution for SES. Two points should be emphasized to ensure that individuals from underserved areas are not denied access to care as medicine moves toward patient satisfaction-based financial models: The importance of risk-adjusted compensation and performance models for orthopaedic surgeons performing RCRs, and second, the importance of preoperative patient expectation counseling.
